# Incidence and Early Mortality of Prosthetic Valve Endocarditis in Patients Undergoing TAVI Compared to SAVR: A Systematic Review and Meta-Analysis

**DOI:** 10.3390/jcm14113866

**Published:** 2025-05-30

**Authors:** Elisa Gastino, Matteo Scarpanti, Alessandro Parolari, Fabio Barili

**Affiliations:** 1 University Unit of Cardiac Surgery, IRCCS Policlinico S. Donato, Università Degli Studi Di Milano, San Donato Milanese, 20097 Milan, Italy; elisa.gastino@gmail.com (E.G.); matteo.scarpanti@gmail.com (M.S.); alessandro.parolari@unim.it (A.P.); 2 Department of Biomedical and Clinical Sciences, Università Degli Studi Di Milano, 20133 Milan, Italy; 3 University Cardiac Surgery Unit, IRCCS Ospedale Galeazzi—Sant’Ambrogio, 20157 Milan, Italy; 4 Department of Epidemiology, Harvard T.H. Chan School of Public Health, Boston, MA 02138, USA

**Keywords:** TAVI, cardiac surgery, endocarditis

## Abstract

**Background**: Transcatheter aortic valve implantation (TAVI) is becoming the most important treatment strategy for aortic valve disease. With its dramatic increase, the rate of major complications and the impact of TAVI on long term outcomes is becoming a pressing issue, especially in terms of comparison with surgical aortic valve replacement (SAVR). PVE is a severe complication that can arise post-procedure, leading to significant morbidity and mortality. The aim of this meta-analysis is to compare the incidence of PVE and 30-day mortality rates between TAVI and SAVR. **Methods**: A comprehensive literature review was conducted, identifying studies that reported the incidence and outcomes of PVE in patients undergoing TAVI and SAVR. The selected studies were assessed for heterogeneity using the χ^2^ test and I^2^ statistic. A random effect model was applied to account for variability among studies. The Odds Ratios (ORs) for 30-day mortality and the incidence of PVE were calculated. Funnel plots were utilized to assess the reliability of the data and potential publication bias. **Results**: The analysis showed no significant difference in 30-day mortality of PVE in TAVI and SAVR, with an OR of 1.29 (CI 0.98–1.69). However, there was a significant difference in the incidence of PVE (HR 0.76, CI 0.61–0.96), with TAVI demonstrating a protective effect attributed to its lesser invasiveness and shorter procedural times. The funnel plots indicated high reliability of the data, with low standard errors and minimal publication bias. **Conclusions**: TAVI and SAVR carry similar 30-day mortality rates for patients with PVE; on the other hand, TAVI shows a lower incidence of PVE due to its minimally invasive nature. These findings suggest that TAVI might be a preferable option for certain patient populations, though further randomized clinical trials are needed to confirm these results and address the limitations of the current study.

## 1. Introduction

Transcatheter aortic valve implantation (TAVI) is establishing itself as the most widely adopted treatment strategy for degenerative aortic valve disease worldwide [1]. In fact, in the last decade, we have observed an exponential growth in the prevalence of patients with aortic valve disease treated with TAVI, with its indication rapidly expanded to now include lower surgical risk and younger patients. As a consequence, from 2002, when the first-in-man TAVI was performed as a compassionate option in an inoperable 57-year-old patient, to this day, TAVI has been performed in over 3 million patients worldwide and in approximately eighty countries [2]. With the increasing number of devices, the rate of long-term complications and its impact on long-term outcomes is becoming a pressing issue [3]. One of the most feared mid to late complications after any prosthetic heart valve replacement is infective endocarditis (IE). Prosthetic valve endocarditis (PVE) accounts for 1% to 5% of all cases of IE, following either transcatheter aortic valve implantation or surgical aortic valve replacement (SAVR), and it has been recognized as the most severe form of IE and is associated with a higher risk of in-hospital mortality [4]. Patients with any prosthetic aortic valve are reported to have an incidence of 0.2 to 1.4 episodes per 100 patient-years, and approximately 1.4% of patients undergoing AVR (aortic valve replacement) develop prosthetic valve IE during the first postoperative year [5]. As more and more devices are implanted, PVE is destined to become an even more frequent clinical entity and a major challenge for the modern cardiac surgeon [5]. The risks related to postoperative PVE vary depending on the time of onset after the procedure and the involvement of surrounding cardiac structures, of which the most common are destruction of the valvular apparatus, pseudoaneurysms and abscesses, fistulas, heart block, and stroke from peripheral embolization of the vegetations [6]. For this reason, PVE requires a multidisciplinary approach and a high level of expertise to clarify the best treatment strategies and reduce mortality [7]. While the treatment strategy for PVE following SAVR is challenging but well established by cardiac surgeons [8], who over the years have encountered a broad spectrum of presentations and issues in cases leading to extensive expertise, especially in high volume centers, PVE after TAVI is a relatively recent complication [3], and it presents its own peculiar characteristics, still leaving some debate on its management [9]. Although TAVI is a less invasive procedure when compared to traditional cardiac surgery, it carries a different burden of potential infections and pathogens, mainly related to the access point for deployment, which is most frequently via the femoral artery [7]. Some more specific risk factors for infections are due to the manipulation of the device itself, as in the case of valve crimping, or hemodynamic/anatomic substrates such as turbulence due to perivalvular leaks, leftover intact/calcified native leaflets, and the frequent presence of further intracardiac prosthetic material (i.e., permanent pacemaker) [10]. Moreover, patients undergoing TAVI, especially prior to the widespread application of this new technique to lower-risk and younger patients, were characterized by higher frailty and/or multiple comorbidities with a higher risk of bacteremia and sepsis, notably significant peripheral vessel disease and diabetes mellitus [11]. On the other hand, SAVR is associated with all the potential infective complications of the surgical access site, which is through the sternum in the vast majority of cases [10]. The potential of bacterial dissemination following deep wound infection poses a serious threat to the implanted prosthesis, leading to the potentially fatal onset of early prosthetic valve endocarditis. Despite the increasing number of studies, throughout the literature, comparing the incidence of PVE after SAVR vs. TAVI, accounting for 0.3–1.2% and 0.6–3.4%, respectively, there are still key points related to their specific characteristics and the treatment planning [10]. According to this, we performed a systematic review and meta-analysis to assess the comparative risk of IE after TAVI and SAVR and the relative early and late mortality, in order to better characterize this relatively new entity and its course.

## 2. Materials and Methods

### 2.1. Search Strategy and Selection Criteria

The study protocol adheres to the Preferred Reporting Items for Systematic Reviews and Meta-analyses (PRISMA) statement (PRISMA checklist). The protocol has been registered in PROSPERO (ID CRD42025630633). We searched publications from MEDLINE, Embase, and the Cochrane Central Register of Controlled Trials (CENTRAL) from 1 January 2016 and 1 January 2025, looking for any papers reporting the incidence of infective endocarditis in patients who were previously subjected to TAVI or surgical aortic valve replacement. To further reduce the probability of losing any major study related to the topic, an electronic search of major cardiothoracic surgery journals in the electronic format (www.clinicaltrials.gov, www.acc.org, www.escardio.org, www.tctmd.com, www.pcronline.com) was performed. Moreover, we included studies from the suggested list of all the applicable studies from the search engine. The title of every article was considered first, and then selected abstracts were searched to identify the peer-reviewed studies reporting the incidence of infective endocarditis in the respective populations of patients undergoing both surgical and transcatheter aortic valve implantation and the relative mortality. The search algorithm is detailed below. Secondary endpoints were defined as all those that did not appear in all articles in our series, such as mortality and follow-up time. In summary, the inclusion criterion was the reporting of the incidence of PVE in the TAVI and SAVR populations in the text or appendix or the presentation of this incidence in selected international meetings. The exclusion criteria ruled out narrative reviews, meta-analyses, and case series/reports. All non-full-text and duplicate publications were excluded, as were articles not written in English. Furthermore, mortality rates and follow-up time were also included in the data-gathering process.

### 2.2. Outcomes Measures

The primary endpoint was the incidence of PVE in the two populations. Secondary endpoints that were considered were 30-day and overall mortality and the total follow-up time of the studies. The effect size was the Risk Ratio for the incidence of PVE and Odds Ratio for mortality.

### 2.3. Data Extraction and Analysis

Two independent investigators (EG and MS) identified articles that fulfilled the pre-specified inclusion criteria. Eligible papers were then reviewed in duplicate, and disagreement was solved by a third investigator (FB). Variables included the following: study name, publication year, study design, number of patients who underwent TAVI and SAVR, incidence of IE in the two groups, in-hospital 30-day mortality, long-term mortality, and overall follow-up time.

### 2.4. Meta-Analysis of Outcomes

Once the manuscripts were identified, we included all the studies comparing the incidence of PVE after TAVI and SAVR. The data were analyzed by a random effect model with Review Manager Version 5.4, RevMan 5 (The Cochrane Collaboration, the Nordic Cochrane Centre, Copenhagen, Denmark). The data could be synthesized only when the number of studies equaled or exceeded two. Measurement data were reported as mean ± standard deviation for continuous variables and frequency with relative percentage for categorical variables. Risk Ratios (RRs) and Odds Ratios (ORs) with standard error were calculated and aggregated on the log scale. Individual and pooled RRs with 95% confidence intervals (CI) were calculated by means of the Der Simonian–Laird method; for Ors, the Durbin–Wu–Hausman test was utilized Hypothesis testing for statistical homogeneity was set at the two-tailed 0.10 level and was based on the Cochrane Q test, with I^2^ values of 25%, 50%, and 75% representing mild, moderate, and severe heterogeneity, respectively [12]. A random effect model was used if I^2^ > 25%; otherwise, a fixed effect model was used. Publication bias was graphically assessed using funnel plots [13].

### 2.5. Risk of Bias and Quality Assessment

The risk of bias was assessed using the Cochrane ROB 2.0 tool and ROBINS-I V2 tool.

### 2.6. Ethics Statement

This meta-analysis study is exempt from ethics approval as we collected and synthesized aggregate data published from previous articles in which informed consent had already been obtained for the individual data by the investigators.

## 3. Results

**Figure 1:**
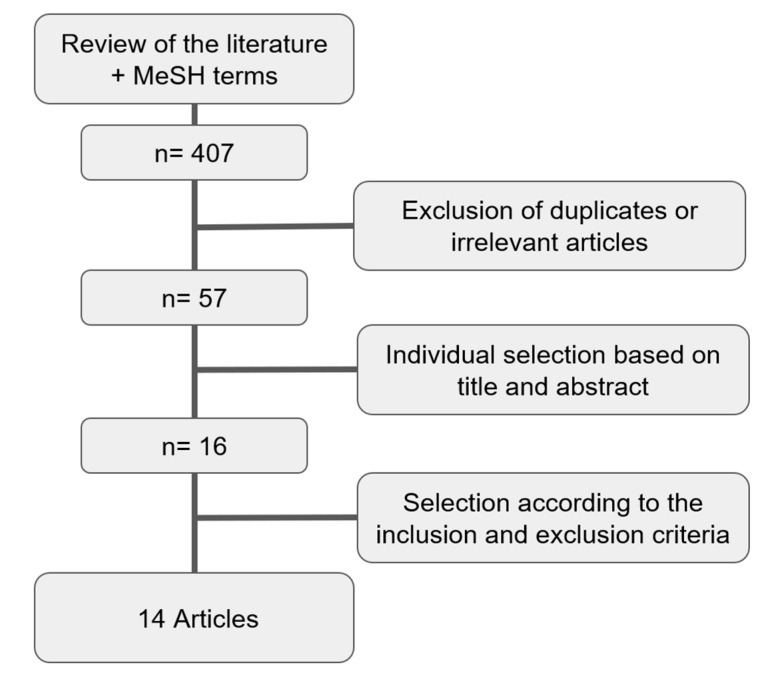
Flowchart for the selection of the studies.

The literature search identified a total of 407 studies. After the analysis of all the abstracts and the exclusion of all irrelevant articles and duplicates, 57 were selected. Once the papers were identified, selection was performed according to the inclusion and exclusion criteria, alongside the availability of full-text files. Works that only reported a single arm (i.e., incidence of IE after either SAVR or TAVI) were also excluded as they had no control group. Two studies that only reported incidences of infective endocarditis without a control population of patients who did not have IE were excluded (Figure 1).

Fourteen articles were finally selected according to the inclusion and exclusion criteria (Table 1).

**Figure 2:**
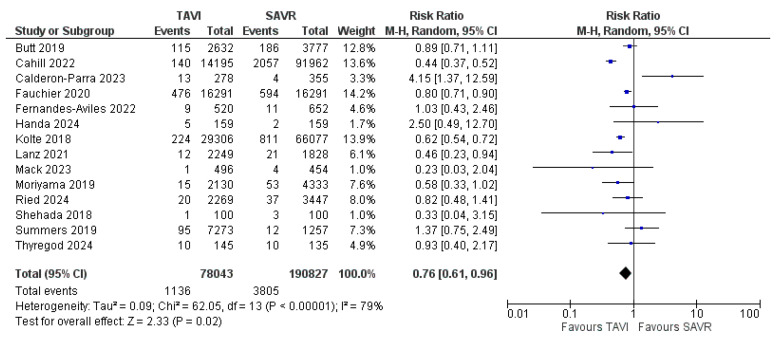
Forrest plot showing the incidence of prosthetic valve endocarditis in TAVI vs. SAVR [14,15,16,17,18,19,20,21,22,23,24,25,26,27].

In total, the review encompassed data from 268,870 patients, of which 4936 were diagnosed with PVE. Of these patients, 190,827 underwent surgical aortic valve replacement and 78,043 received TAVI, of which 3801 and 1135 had prosthetic valve endocarditis, respectively. The articles spanned seven years and included seven retrospective observational studies (ROBs), three propensity-score-matched studies (PSMs), three randomized clinical trials (RCTs), and one prospective observational study (POB). Table 1 shows the studies included and the variables utilized in the analysis. The primary outcome in terms of the incidence of infective endocarditis in SAVR and TAVI showed a dramatic difference in the data reported throughout the literature comparing the two populations of patients (Figure 2). The Risk Ratio (RR) for the primary outcome was 0.76 [CI 0.61–0.96] with a test for the overall effect Z = 2.33 (*p* = 0.02), thereby highlighting a statistically significant difference in terms of incidence of prosthetic valve endocarditis in TAVI and SAVR, with a clearly favorable outcome for TAVI.

**Figure 3:**
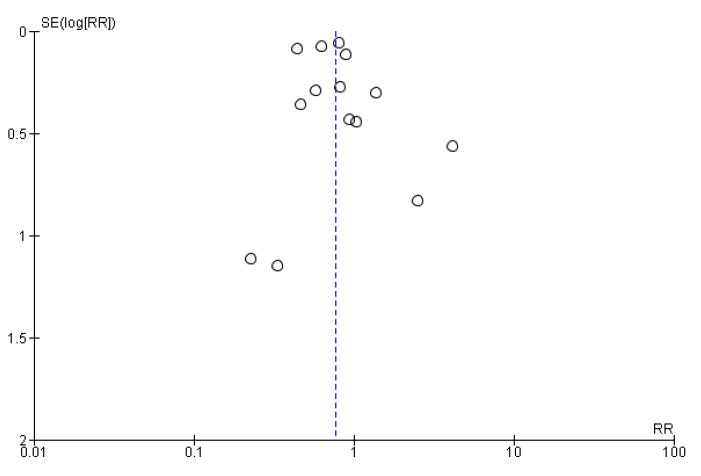
Funnel plot showing the distribution of the reliability of the studies.

Moreover, the sample size is adequate in terms of populations, and all tests for heterogeneity allow for a random effect model to be applied (Heterogeneity was calculated at a τ^2^ of 0.09, with a χ^2^ of 62.05 and an I^2^ of 79%.). The funnel plot (Figure 3) visually shows the high reliability of almost all data, with the SE being very low and all points being centered around an RR of 1.

**Figure 4:**
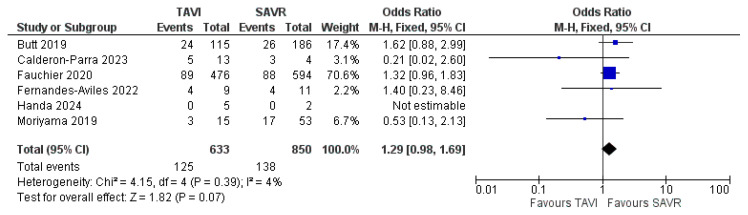
Forest plot showing 30-day mortality with PVE in TAVI vs. SAVR [14,16,17,18,19,23].

**Figure 5:**
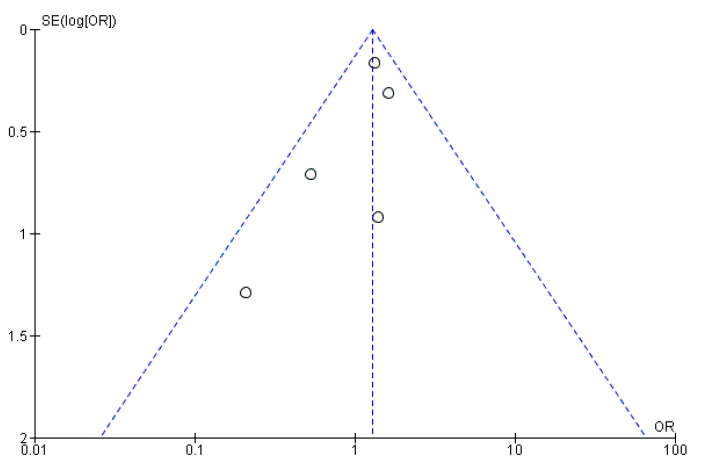
Funnel plot showing the distribution of the studies assessing 30-day mortality.

**Figure 6:**
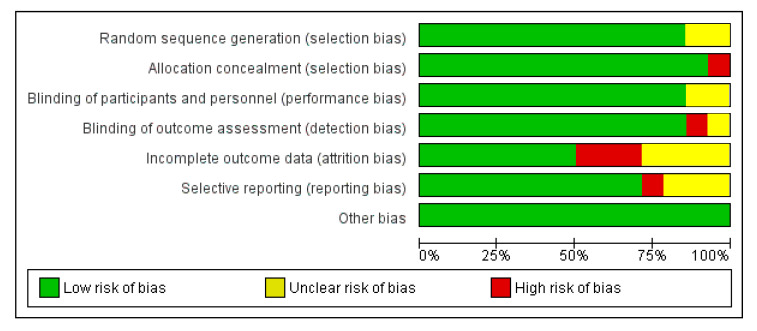
Risk of bias assessment.

An analysis of mortality rates reported throughout the studies showed no significant difference between PVE in TAVI and PVE in SAVR in terms of 30-day mortality, with an Odds Ratio (OR) of 1.29 (CI 0.98–1.69) and a test for the overall effect of 1.82 (*p* = 0.07). These results come from remarkably fewer studies and, therefore, a much smaller sample size. Due to low heterogeneity (χ^2^ of 4.15 and I^2^ of 4%), a fixed effect model was used. The ORs and a forest plot are shown in Figure 4. Moreover, a visual assessment of the funnel plot showed an adequate distribution of the studies (albeit much fewer in number), as shown in Figure 5. A complete bias assessment was performed by the authors, and it is shown in Figure 6.

## 4. Discussion

Our findings across the widest selection of articles reporting the incidence of prosthetic valve endocarditis in patients who underwent TAVI and SAVR show a significant difference between the two populations [20]. The protective effect of TAVI on the incidence of PVE could be attributed to lesser invasiveness and quicker procedural times. In addition, many of the patients undergoing transcatheter aortic valve implantation usually have a shorter hospital stay; they do not necessitate intensive care management and, therefore, may be less likely to be subjected to nosocomial pathogens [25]. On the other hand, the in-hospital mortality rates of patients with PVE associated with TAVI are higher than those with SAVR (albeit not statistically significant), likely due to the higher inherent frailty of these patients, who may be deemed to be at an excessively high risk for any future surgical treatment [23]. This may represent a form of under-treatment of a population of patients who were likely allocated to a less invasive procedure because of their comorbidities and frailty, not necessarily just their age. This hypothesis is further supported by the fact that all the data on 30-day mortality of PVE come from retrospective studies [14,20,26], not from randomized clinical trials; therefore, the equipoise of patient selection cannot be applied.

These results could have several important implications for clinical practice, particularly in the patient selection process and risk stratification. Moreover, the findings highlight the importance of infection control measures that should be considered in both TAVI and SAVR procedures. For what concerns TAVI procedures, even with a lower incidence of nosocomial infections, clinicians should be aware of the specific risk factors and implement targeted strategies to reduce the in-hospital mortality rates associated with the frailty of TAVI patients and also the invasiveness of a surgical TAVI explant. Given the complexity of SAVR and TAVR PVE, a multidisciplinary approach involving cardiac surgeons, cardiologists, internists, and infectious disease specialists should be recommended to improve the quality of the treatment plan, from the preoperative assessment and risk stratification to the surgical approach selection and complication management. This suggests the relevance of the current topic of personalized medicine, considering the individual characteristics of patients undergoing aortic valve replacement, such as comorbidities, frailty, and risk factors for infections. This personalized approach can ensure the most appropriate and effective treatment for each patient.

This study has some inherent limitations. Because of the nature of the papers taken into consideration and the system of data reporting in retrospective studies, there is an intrinsic bias in terms of the primary endpoint. The only data that were considered for analysis were taken from studies that compared the incidence rates and follow-up of patients undergoing transcatheter aortic valve implantation and surgical aortic valve replacement in the same setting. The majority of the studies are retrospective observational studies, and therefore, they have little to no information on the preoperative determinants that led to the percutaneous versus surgical strategy. This allocation can represent a significant confounding factor when considering that a non-negligible number of patients underwent surgical aortic valve replacement for infective endocarditis in the first place, and these are at an absolute higher risk of developing recurrences as well as new-onset PVE. For this reason, the most reliable data are those coming from RCTs and PSMs, which ideally both should have a 0% IE in the SAVR group. Another aspect to consider is the heterogeneity calculated on the forest plot showing the incidence of prosthetic valve endocarditis in TAVI vs. SAVR, which led to further analysis showing that the outliers did not affect the result, although they significantly reduced heterogeneity. This study’s limitations highlight the need for further research focusing on prospective randomized trials or PSMs to provide more robust evidence for clinical decision making. Moreover, research should explore the long-term outcomes after TAVI and SAVR, including the impact on quality of life and functional status.

Under-reporting of incidence data may play a significant role, as the patients can often be admitted and diagnosed with IE at an institution different from where the patients received the bioprosthetic valves in the first place (except for data from randomized clinical trials in which patients ought to receive accurate follow-up from the investigators) and therefore represent a substantial percentage of loss to follow-up. This is particularly relevant in the population of patients in which a potential surgical explant of the infected prosthesis is excluded due to frailty or significant comorbidities. Moreover, the results, despite the presence of two studies with significantly larger populations, can be regarded as accurate, as shown by the forest plot in Figure 2. In summary, the results of this meta-analysis suggest that TAVI may offer a lower risk of PVE compared to SAVR, making it a potentially preferable option for certain patient populations. However, the higher in-hospital mortality rates associated with PVE in TAVI patients highlight the need for careful patient selection, vigilant infection control, and a multidisciplinary approach to care.

## 5. Conclusions

From the results of the present meta-analysis, it is evident that the reported rates of infective endocarditis following surgical aortic valve replacement and transcatheter aortic valve implantation are very different, with a statistically lower incidence in TAVI and comparable mortality with PVE affecting surgically implanted valves.

## Figures and Tables

**Table 1:** The list of studies and relative data.

N#	Author	Year	Journal	Article Type	PVE TAVI	Tot. TAVI	PVE SAVR	Tot. SAVR	30-Day Mort. TAVI (%)	30-Day Mort. SAVR (%)	Mort. TAVI (%)	Mort. SAVR (%)	FU (m)
1	Butt [14]	2019	*JACC*	ROB	115	2632	186	3777	24 (20.9)	26 (13.9)	N/A	N/A	1
2	Cahill [15]	2022	*Heart*	ROB	140	14,195	2057	91,962	N/A	N/A	N/A	N/A	N/A
3	Calderon-Parra [16]	2023	*J. Clin. Med.*	PSM	13	278	4	355	5 (38.5)	3 (75)	7 (53.8)	3 (75)	12
4	Fauchier [17]	2020	*Clinical Microbiology and Infection*	PSM	476	16,291	594	16,291	89 (18.7)	88 (14.8)	156 (32.8)	179 (30)	12
5	Fernandez-Aviles [18]	2022	*Clinical Cardiology*	ROB	9	520	11	652	4 (44.4)	4 (36.4)	4 (44.4)	4 (36.4)	1
6	Handa [19]	2024	*Ann. Thorac. Cardiovasc. Surg.*	PSM	5	159	2	159	0 (0%)	0 (0%)	N/A	N/A	86
7	Kolte [20]	2018	*Am. J. Cardiol.*	ROB	224	29,306	811	66,077	35 (15.6)	N/A	35 (15.6)	N/A	10
8	Lanz [21]	2021	*JAHA*	RCT	12	2249	21	1828	N/A	N/A	N/A	N/A	12
9	Mack [22]	2023	*NEJM*	RCT	1	496	4	454	N/A	N/A	N/A	N/A	60
10	Moriyama [23]	2019	*EuroIntervention*	ROB	15	2130	53	4333	3 (20)	17 (32)	3 (20)	17 (32)	1
11	Ried [24]	2024	*Clin Res Cardiology*	ROB	20	2269	37	3447	N/A	N/A	6 (30)	10 (27)	12
12	Shehada [25]	2018	*Journal of Thoracic* *Disease*	POB	1	100	3	100	N/A	N/A	N/A	N/A	24
13	Summers [26]	2019	*Circulation*	ROB	95	7273	12	1257	N/A	N/A	N/A	N/A	60
14	Thyregod [27]	2024	*EHJ*	RCT	10	145	10	135	N/A	N/A	N/A	N/A	120

Abbreviations: ROB—retrospective observational; PSM—propensity-score-matched; RCT—randomized clinical trial; PVE—prosthetic valve endocarditis; TAVI—transcatheter aortic valve implantation; FU—follow-up.

## Data Availability

No new data were created or analyzed in this study. Data sharing is not applicable to this article.

## Abbreviations

The following abbreviations are used in this manuscript:

CI	Confidence interval
HR	Hazard ratio
OR	Odds Ratio
RR	Risk Ratio
SAVR	Surgical aortic valve replacement
TAVI	Transcatheter aortic valve implantation
PVE	Prosthetic valve endocarditis
